# Integrated High-Throughput Sequencing, Microarray Hybridization and Degradome Analysis Uncovers MicroRNA-Mediated Resistance Responses of Maize to Pathogen *Curvularia lunata*

**DOI:** 10.3390/ijms232214038

**Published:** 2022-11-14

**Authors:** Weiwei Wang, Zhen Liu, Xinyuan An, Yazhong Jin, Jumei Hou, Tong Liu

**Affiliations:** 1 Key Laboratory of Green Prevention and Control of Tropical Diseases and Pests, Ministry of Education, Hainan University, Haikou 570228, China; 2 Key Laboratory of Genetics and Germplasm Innovation of Tropical Special Forest Trees and Ornamental Plants, Ministry of Education, Hainan University, Haikou 570228, China; 3 College of Agriculture, Heilongjiang Bayi Agricultural University, Daqing 163319, China

**Keywords:** disease resistance, Huangzao 4, Luyuan, PC-732, PC-169, stem-loop RT-PCR

## Abstract

*Curvularia lunata* (Wakker) Boed, the causal agent of leaf spot in maize, is prone to mutation, making it difficult to control. RNAi technology has proven to be an important tool of genetic engineering and functional genomics aimed for crop improvement. MicroRNAs (miRNAs), which act as post-transcriptional regulators, often cause translational repression and gene silencing. In this article, four small RNA (sRNA) libraries were generated from two maize genotypes inoculated by *C. lunata*; among these, ltR1 and ltR2 were from the susceptible variety Huangzao 4 (HZ), ltR3 and ltR4, from the resistant variety Luyuan (LY), and 2286, 2145, 1556 and 2504 reads were annotated as miRNA in these four sRNA libraries, respectively. Through the combined analysis of high-throughput sequencing, microarray hybridization and degradome, 48 miRNAs were identified as being related to maize resistance to *C. lunata*. Among these, PC-732 and PC-169, two new maize miRNAs discovered, were predicted to cleave mRNAs of metacaspase 1 (*AMC*1) and thioredoxin family protein (*Trx*), respectively, possibly playing crucial roles in the resistance of maize to *C. lunata*. To further confirm the role of PC-732 in the interaction of maize and *C. lunata*, the miRNA was silenced through STTM (short tandem target mimic) technology, and we found that knocking down PC-732 decreased the susceptibility of maize to *C. lunata*. Precisely speaking, the target gene of PC-732 might inhibit the expression of disease resistance-related genes during the interaction between maize and *C. lunata*. Overall, the findings of this study indicated the existence of miRNAs involved in the resistance of maize to *C. lunata* and will contribute to rapidly clarify the resistant mechanism of maize to *C. lunata*.

## 1. Introduction

Maize (*Zea mays*), one of the most important cereal crops, is widely grown throughout the world and used as one of the most important staple foods worldwide. However, leaf spot disease caused by *Curvularia lunata* (Wakker) Boed has led to great yield losses in maize growing areas of the world in the past decades. At present, the resistant varieties were mainly used to control this disease in China, for example ‘shenshi29’, ‘danyu24’ and ‘liaodan933’. However, some evidence has indicated that the pathogen has a high degree of pathogenicity variation, suggesting that the disease may have the possibility of outbreak [1,2,3]. It is difficult and time-consuming for conventional breeding to deal with the situation. Therefore, it is of great significance to clarify the interaction mechanism between maize and *C. lunata* to effectively control the disease.

The genetic pattern of resistance to *C. lunata* in maize was found to be based on quantitative inheritance, indicating that multiple genes may be involved in the host resistance [4]. With the advancements of molecular biology and proteomics, great progress has been made in the research of molecular resistance mechanism. The quantitative trait loci (QTL) associated with resistance to *C. lunata* in maize were identified [5]. Furthermore, eight proteins associated with host defense response were found in maize by comparative proteomics, and were identified as germin-like protein GLP and translation initiation factor Eif-5A, which may play important roles in maize resistance against *C. lunata* infection [4]. However, how the genes or proteins are involved in the resistance regulation of maize against *C. lunata* is still elusive.

RNA interference (RNAi), a gene silencing phenomenon, has evolved in recent years as a vital tool of genetic engineering and functional genomics aimed for crop improvement [6]. The microRNAs (miRNAs) are small, endogenous, single-stranded and ~21 nt long RNAs and have been identified as important regulators of gene expression in many plants [6,7]. Several recent publications have suggested that miRNA-mediated gene silencing may serve as a general regulatory mechanism in plant immune responses to pathogens [8,9]. Members of a miRNA family comprising miRNAs with the same seed region (positions 2–8 of the mature miRNA) and highly similar secondary structure often have overlapping targets allowing for more robust repression of target pathways [10]. For example, miR393 can negatively regulate the mRNA for F-box auxin receptors TIR1, AFB2 and AFB3, which contributes to plant resistance against bacteria, whereas miRNA398b and miR773 negatively regulate plant disease resistance [11,12,13].

Recently, many miRNAs relative to plant defense response have been identified in many species through high-throughput sequencing and microarray hybridization [14,15,16,17]. The expressional patterns of miRNAs upon infection by *Phytophthora sojae* were examined by microarray analysis [18]. miR169, miR160 and miR171a were identified using microarray analysis during the interaction between tomato and *Botrytis cinerea* [19]. In *Exserohilum turcicum*-inoculated leaves, 118 miRNAs were detected and miR811 and miR829 were found to confer a high degree of resistance to *E. turcicum* [20]. As more and more miRNAs are discovered, the determination of miRNA targets has become the key to understand the biological function of miRNAs. Degradome sequencing, referring to uncoupling the sliced subset of miRNA targets from a priori computational target predictions, combines the advantages of high-throughput sequencing, bioinformatics and RACE, and has been widely used to search the targets of miRNAs [21]. This technology has been used successfully in *Arabidopsis thaliana* [22], *Oryza sativa* [23], *Physcomitrella patens* [21], *Vitis vinifera* [24], *Glycine max* [25], *Zea mays* [26] and *Cucumis sativus* [27] among other studies to identify targeted genes of miRNAs.

Currently, many miRNAs have been identified in maize by high-throughput sequencing and computational analysis [28]. Some miRNAs were induced under environmental stress, indicating that miRNAs played critical and different roles in the regulation of metabolic, developmental processes and adaptation to stress [28]. However, no miRNA associated with the resistance of maize to *C. lunata* was reported. In this article, a large number of miRNAs that were involved in resistance response were identified by high-throughput sequencing, the response process was visualized by the microarray hybridization technique, and the corresponding target genes of miRNAs were confirmed by degradome analysis. The findings may clarify a new resistant mechanism of maize to *C. lunata* and can promote the application and popularization of miRNAs in maize breeding of resistant cultivars.

## 2. Results

### 2.1. Overview of sRNA Libraries 

#### 2.1.1. Characteristics of Four sRNA Libraries

**Figure 1:**
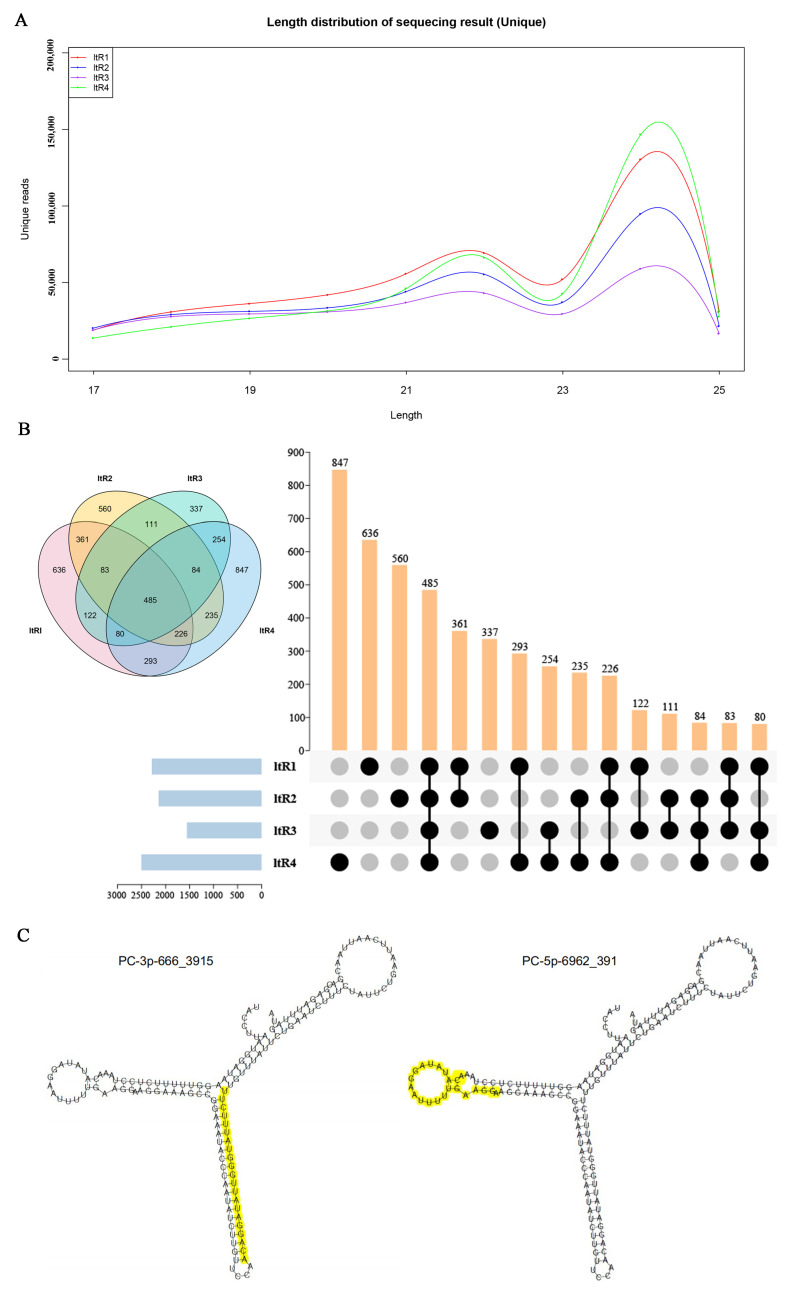
Overview of four sRNA Libraries. (**A**) The length distribution of small RNAs in the four libraries. (**B**) Venn diagram of the number of miRNAs expressed in the four sRNA libraries. “Blue columns” represent the total number of miRNAs in each library; “orange columns” represent the number of miRNAs expressed in different libraries; “black dots” represent that the miRNAs could be expressed in the library listed in the left and “grey dots” represent that the miRNAs could not be expressed in the library listed in the left. (**C**) The secondary structure of two miRNAs derived from different arms of the same precursor. Sequences marked in yellow represented mature miRNA.

The susceptible variety HZ and the resistant variety LY were infected by *C. lunata* (Figure S1) and sampled to construct four sRNA libraries, which were named after ltR1, ltR2, ltR3 and ltR4, respectively. In total, the numbers of raw reads were 1,050,117, 910,208, 848,756 and 1,009,022 for these four libraries, respectively (Table 1 and Table S3). Clean reads were used for further analysis. After deduplication, the length of the majority of sRNA reads over all libraries varied from 17 nt to 25 nt and the abundance of sRNAs with each length was different (Figure 1A). 21 nt to 24 nt were the most abundant, although not in equal quantity in all the libraries. Among these, the most frequent was 24 nt (27.97% for ltR1, 25.88% for ltR2, 20.21% for ltR3 and 34.72% for ltR4) (Figure 1A). In the four libraries, 2286 (0.22%), 2145 (0.24%), 1556 (0.18%) and 2504 (0.25%) were annotated as miRNA, respectively (Table 1 and Table S3). A total of 485 miRNAs were co-expressed, while 636, 560, 337 and 847 miRNAs were specifically expressed in each library, respectively (Figure 1B). The number of miRNAs expressed specifically in ltR4 was noticeably higher (847 miRNAs in ltR4) than other three libraries.

#### 2.1.2. Identification of Known MiRNAs

In total, 454 known miRNAs were identified from the four sRNA libraries. Among these, 443 miRNAs belonging to 120 families were conserved in plants and 11 miRNAs belonged to 4 families were non-conserved (Table S4). For example, miRNA156, miRNA159, miRNA160, miRNA164, miRNA166, miRNA167, miRNA169, miRNA171, miRNA395, miRNA396 and miRNA399 were conserved miRNAs, while miRNA390, miRNA393, miRNA394 and miRNA529 were non-conserved and only found in maize. In addition, we found that most of the conserved miRNA families contained only one member, but others were multi-member families, such as miRNA156, miRNA169, miRNA166, miRNA167, miRNA159/171 and miRNA395, including 35, 32, 29, 26, 25 and 20 members, respectively. Meanwhile, the expression level of different members in the same family also showed significant difference. For example, the normalized read count of family miR166 ranged from 0 to 2579 in the four libraries, and 0 to 3786 for miR168. Furthermore, the same member within different sRNA libraries also showed different expression levels, for example the normalized read count of zma-miR156a-5p in ltR1, ltR2, ltR3 and ltR4 were 407, 736, 357 and 255, respectively. The vast expression differences among different members within the same family or within different families could indicate that miRNAs might be related to the disease-resistant response of maize to *C. lunata*.

#### 2.1.3. Identification of Novel MiRNAs

In total, 72 putative novel miRNAs were identified, in which 51 were conserved miRNAs originating from 28 miRNAs families and 21 were non-conserved miRNAs in 9 miRNAs families. Moreover, 4 new miRNAs that did not belong to the above families were also identified, and they were PC-5p-6962_391, PC-3p-666_3915, PC-3p-836_2992 and PC-5p-12301_242 (Table S5). The members of novel miRNA families were fewer compared to some known conserved miRNA families, for example, the family member MIR171 was the one with more members, although this family only had 8 miRNAs. Most novel miRNAs were induced or specific in treatment (ltR2 or ltR4) compared with control (ltR1 or ltR3), except MIR397, MIR1310 and MIR2916. For example, the expression of zma-MIR166k-p5_1ss6AT, bdi-MIR159b-p5 and sbi-MIR1432-p3_1ss21AT was induced in the susceptible variety HZ, and sbi-MIR156c-p3, sbi-MIR171h-p5 and sbi-MIR437g-p3_1ss3AG were induced in the resistant variety LY.

Interestingly, zma-MIR319a-p5 and zma-MIR319c-p5, belonging to the family MIR319, had the same sequences, but derived from different precursors. The genome ID of precursor of zma-MIR319a-p5 was gi414875515, but it was gi408831849 for zma-MIR319c-p5. This phenomenon was also found in the MIR528 family. The precursors of two new miRNAs (osa-MIR5079a-p5_1ss20TC and osa-MIR5079a-p3_1ss12CT) belonging to MIR5079 came from the same locus in the maize genome. Moreover, the secondary structures of PC-3p-666_3915 and PC-5p-6962_391 showed that they might derived from different arms of the same precursor (Figure 1C), which suggested that most pre-miRNAs were cleaved in different sites in the process of mature miRNA production.

Furthermore, 3301 sRNAs unique sequences, which cannot be mapped in miRBase but were complementary to maize genome, were found to be as potential novel miRNAs of maize. These miRNAs were named starting with the letters PC, such as PC-5p-531639 (Table S6).

#### 2.1.4. Analysis of MiRNA Family

In total, 72 miRNA families were identified through miRNA family analysis (Table S7). Some miRNA families were high abundant. For example, the number of members in MIR159 was the most, including 29 members, followed by miR166, miR156, miR171_1, miR399, miR167_1, which contained 26, 23, 18, 17 and 16 members, respectively. However, most miRNA families were less abundant, and only 1 or 2 members were included. Further, three new miRNA families were found in maize, which were miR437, miR2275 and miR5079, including osa-MIR437-p3_1ss9GA and sbi-MIR437g-p3_1ss3AG, zma-MIR2275b-p5, osa-MIR5079a-p5_1ss20TC and osa-MIR5079a-p3_1ss12CT, respectively.

### 2.2. MiRNAs Responsive to C. lunata Identified by Microarray

To investigate the expression profiles of miRNAs in the susceptible variety HZ and the resistant variety LY infected by *C. lunata*, microarray was performed, in which 12 chips were produced, and 1079 probes were used for each chip. According to the results, many miRNAs showed significant alterations in expression in response to *C. lunata* infection. In summary, 176, 190 and 153 miRNAs were identified to be responsive to *C. lunata* in HZ at 3, 9 and 15 hpi, respectively (*p*-value < 0.01 and ∣log_2_ fold change∣x > 1.5; Table S8), and in LY, 103, 132 and 227 miRNAs were responsive to *C. lunata* at 3, 9 and 15 hpi, respectively (*p*-value < 0.01 and ∣log_2_ fold change∣> 1.5; Table S9). The number of miRNAs differentially expressed at each inoculation time in HZ or in LY was different. In HZ, the highest number was at 9 hpi, while in LY, occurred at 15 hpi.

**Figure 2:**
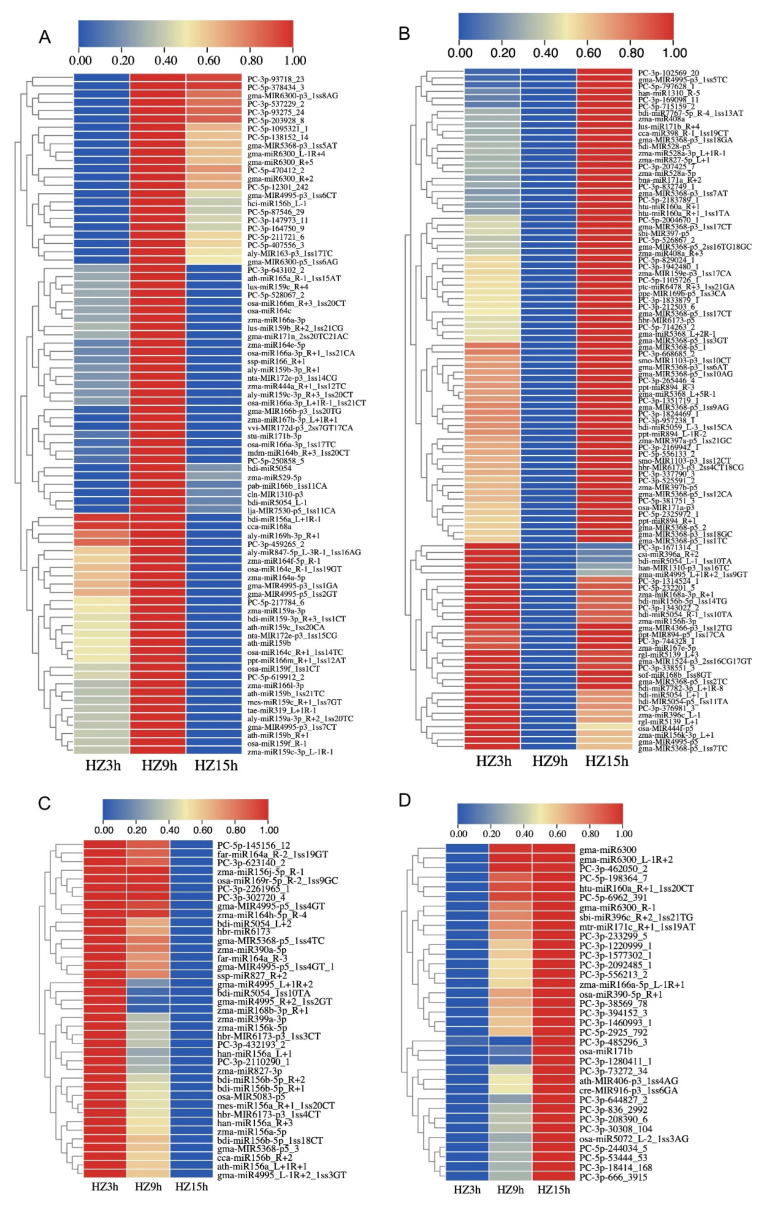
Level of expression of miRNAs in response to *C. lunata* in the susceptible variety HZ at different inoculation time points (3, 9 and 15 hpi). The expression trends were divided into four categories: first increase and then decrease (**A**), first decrease and then increase (**B**), reduced expression (**C**) and increased expression (**D**).

**Figure 3:**
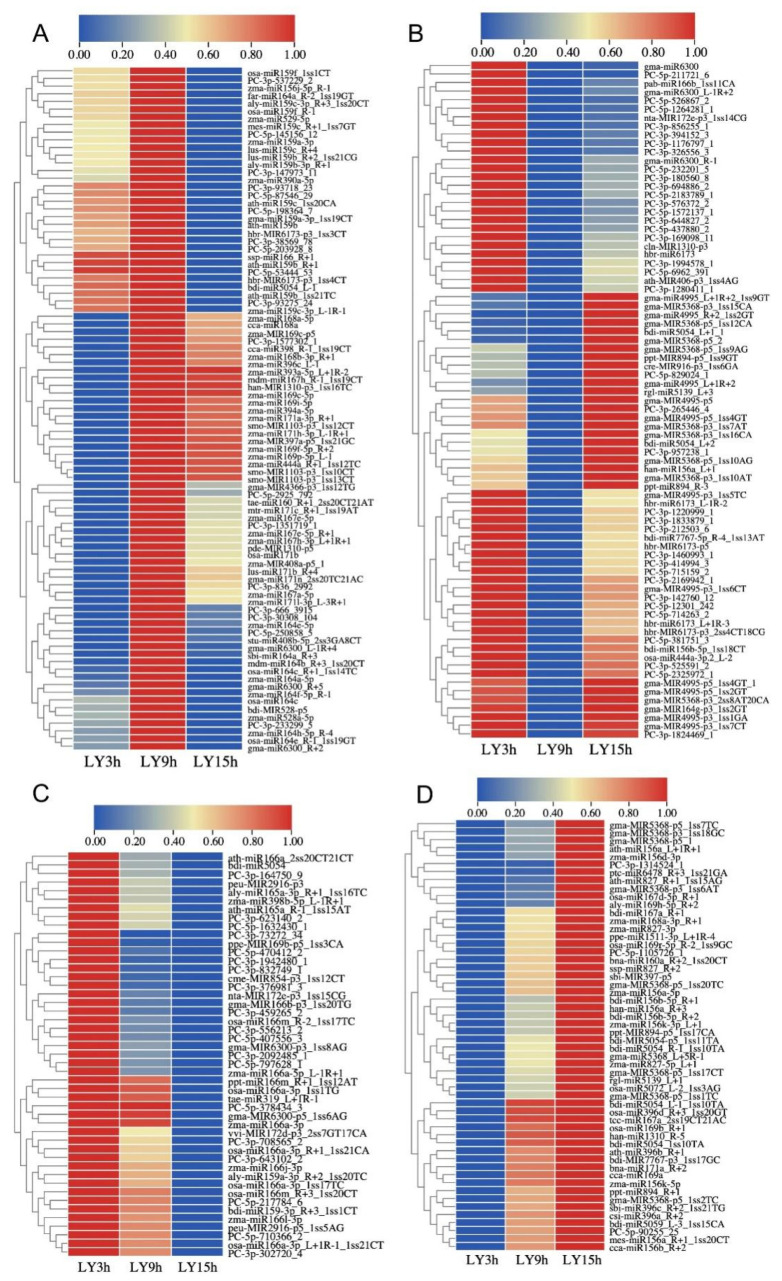
Expression dynamics of miRNAs in response to *C. lunata* in the resistant variety LY at different inoculation time points (3, 9 and 15 hpi). The expression trends were divided into four categories: first increase and then decrease (**A**), first decrease and then increase (**B**), reduced expression (**C**) and increased expression (**D**).

To examine the expression trends of miRNAs which were responsive to *C. lunata* in the interaction with maize (HZ and LY) and were detectable at all the three inoculation time points (3, 9 and 15 hpi), cluster analysis was carried out, and part of miRNAs meeting the above-mentioned requirements were selected to do the analysis. As shown in the cluster analysis, the expression trends of miRNAs could be divided into four categories: first increase and then decrease (ID), first decrease and then increase (DI), reduced expression (D) and increased expression (I) (from 3 to 15 hpi). The number of miRNAs following the above four categories were 82 (31.78%), 102 (39.53%), 39 (15.12%) and 35 (13.57%) in HZ (Figure 2), and 89 (33.09%), 79 (29.37%), 47 (17.47%) and 54 (20.07%) in LY (Figure 3). Meanwhile, there were 69 and 134 miRNAs whose expression levels changed significantly when compared with the control in HZ and in LY, respectively (*p*-value < 0.01 and ∣log_2_ fold change∣>1.5; Figure S2).

Furthermore, the comparative expression levels analysis of miRNAs between HZ and LY responsive to *C. lunata* found 148 miRNAs (*p*-value < 0.01 and∣log_2_ fold change∣> 1.5) that were differentially expressed (Figure S3 and Table S10). For example, some members of miR5368 and miR6300 were down-regulated, while some members of miR164 and miR171 were up-regulated in LY compared with the pattern of expression in HZ.

### 2.3. Target Genes of MiRNAs in Maize Searched through Degradome Analysis

**Figure 4:**
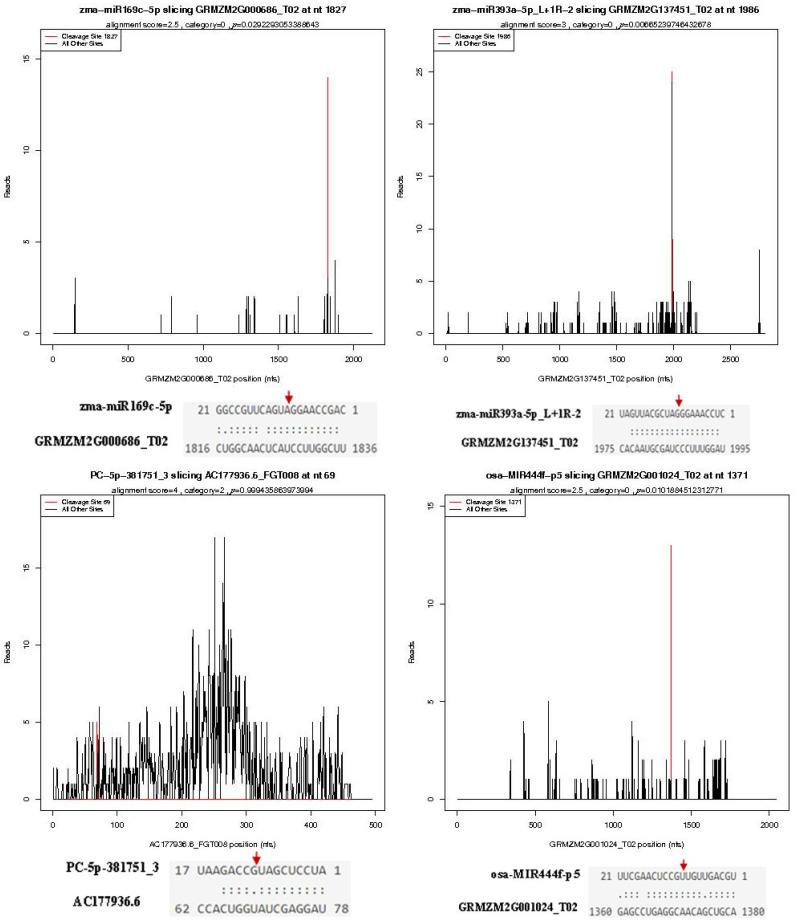
T-plot of miRNAs and their corresponding target genes. The red arrows indicate the splicing sites of the target gene to miRNA.

To search the targets of identified miRNAs, two independent degradome libraries of susceptible variety HZ and resistant variety LY were constructed and high-throughput sequenced. A total of 11,473,928 and 13,961,496 clean reads were obtained in the HZ and LY degradome libraries, respectively, of which 9,517,279 (82.62%) and 11,676,151 (83.33%) matched to the maize transcriptome (Table 2). Through TargetFinder, a total of 13,026 mRNAs were predicted to be targeted by 1013 miRNAs (Table S11). Among these miRNAs, 665 miRNAs were corroborated to cleave 1584 targets sequenced in the degradome (Table S12). The cleavage sites for some miRNA–mRNA alignments are shown in Figure 4. The cleavage sites of most miRNAs were between 10 and 11 of the targets. Unfortunately, the target genes of some identified miRNAs, especially novel ones, could not be detected in the present degradome libraries. For example, PC-3p-104754_19, PC-3p-73272_34, PC-3p-1280411_1 and PC-3p-130200_14, were predicted to cleave 25, 13, 11, 6 targets, respectively, but no targeted mRNAs were found in degradome libraries.

**Figure 5:**
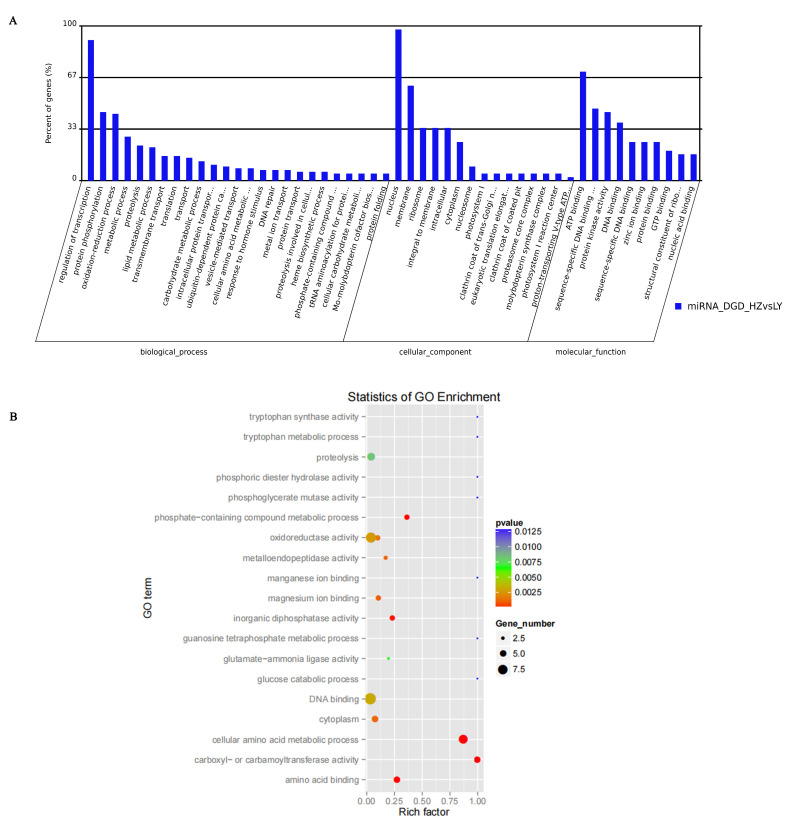
Function analysis of targets of miRNAs expressed differentially. (**A**) Functional clustering analysis of target genes of miRNAs expressed differentially significantly. (**B**) GO analysis of targets of miRNAs expressed differentially at different inoculation times by *C. lunata*.

The functions of targets paired with miRNAs that were differentially expressed were annotated through GO analysis (http://www.geneontology.org/) (accessed on 25 October 2021). Most of the target genes participated in “regulation of transcription”, “protein phosphorylation” and “oxidation-reduction process” within the category “biological process”; participated in “nucleus” and “membrane” within category “cellular component”; and participated in “ATP binding”, “sequence-specific DNA binding transcription factor activity” and “protein kinase activity” within category “molecular function” (Figure 5A). Through GO enrichment, their functions were mainly involved in oxidoreductase activity, DNA binding, cellular amino acid metabolic process, carboxyl- or carbamoyltransferase activity, amino acid binding, phosphate-containing compound metabolic process and proteolysis, among other functions (Figure 5B). According to the gene annotations, members of the same miRNA family often cleaved the same mRNA and were involved in the same biological process (Table S12). For example, the target genes of miR159, miR396, miR164, miR169 and miR171 were annotated as myb domain protein 65, growth-regulating factor 2/5, NAC domain containing protein, nuclear factor Y and GRAS family transcription factor, respectively.

To test if the expressions of miRNAs and their corresponding targets were negatively correlated, stem-loop RT-PCRs were examined in both maize genotypes. According to results of microarray and degradome sequencing, five miRNAs were selected, including three known miRNAs (zma-miR169c-5p, zma-miR393a-5p_L+1R-2 and zma-miR164e-5p) and two novel miRNAs (PC-3p-73272_34 and PC-3p-169098_11), and their corresponding targets were nuclear factor Y (*NY*), auxin signaling F-box 2 (*AFB*2), NAC domain containing protein 80 (*NAC*), metacaspase 1 (*AMC*1) and thioredoxin family protein (*Trx*), respectively.

**Figure 6:**
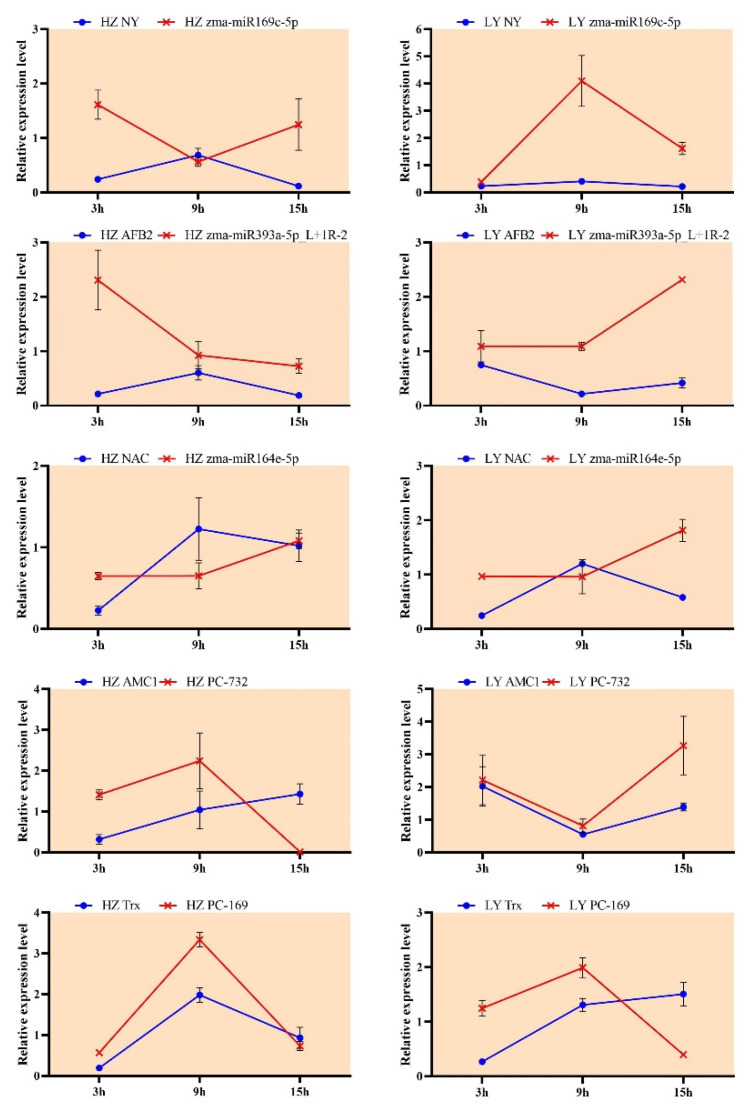
The expression pattern of 5 miRNAs and their targets in HZ (**left**) and LY (**right**) infected by *C. lunata*. zma-miR169, zma-miR393, zma-miR164, PC-732 and PC-169 were abbreviated names of miRNAs, and their full names were zma-miR169c-5p, zma-miR393a-5p_L+1R-2, zma-miR164e-5p, PC-3p-73272_34 and PC-3p-169098_11, respectively.

At 15 hpi, the miRNA zma-miR169 was expressed up-regulated and the expression of its target mRNA *NY* was down-regulated in the susceptible variety HZ; at 3 hpi, the expression of zma-miR393 was up-regulated, while the expression of *AFB*2 was down-regulated in HZ; at 15 hpi, the miRNA zma-miR164 negatively regulated the expression of *NAC* in the resistant variety LY (Figure 6). Similarly, the negative regulation could also be observed from PC-732 (PC-3p-73272_34)/*AMC*1 at 9 hpi in HZ and from PC-169 (PC-3p-169098_11)/*Trx* at 15 hpi in LY. The results showed that the expression pattern of miRNAs and their corresponding target genes were negative relationship.

### 2.4. MiRNAs Associated to Disease Resistance Identified through Combined Analysis of High-Throughput Sequencing, Microarray Hybridization and Degradome

**Figure 7:**
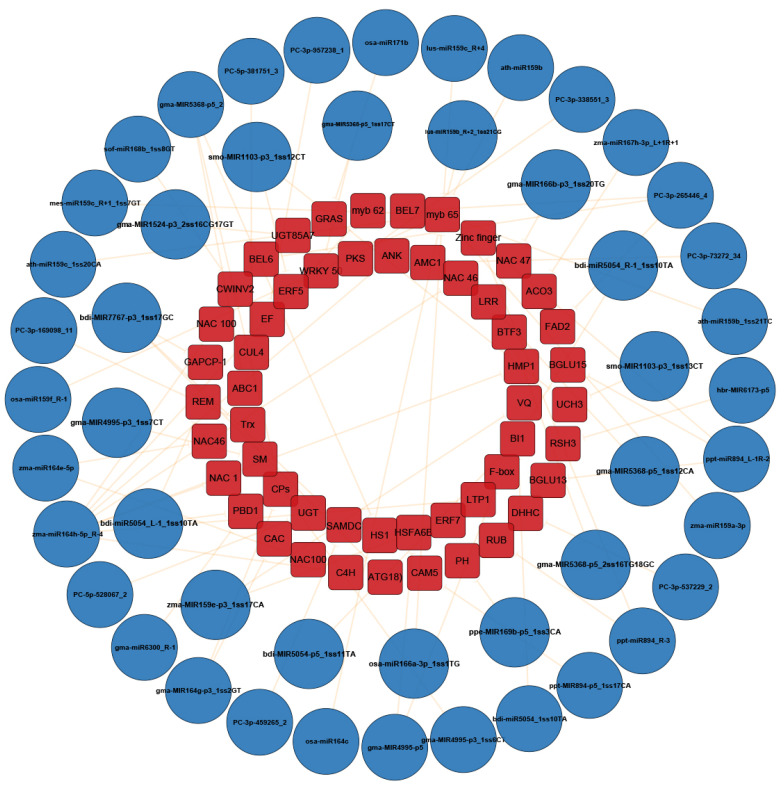
An interaction network indicating the relationship between miRNAs associated with the disease resistance of maize to *C. lunata* and their corresponding target genes. The blue circles represent the miRNAs and red rectangles represent the target genes. The characters in the red rectangles stand for the abbreviation of target genes showing in Table S13.

**Figure 8:**
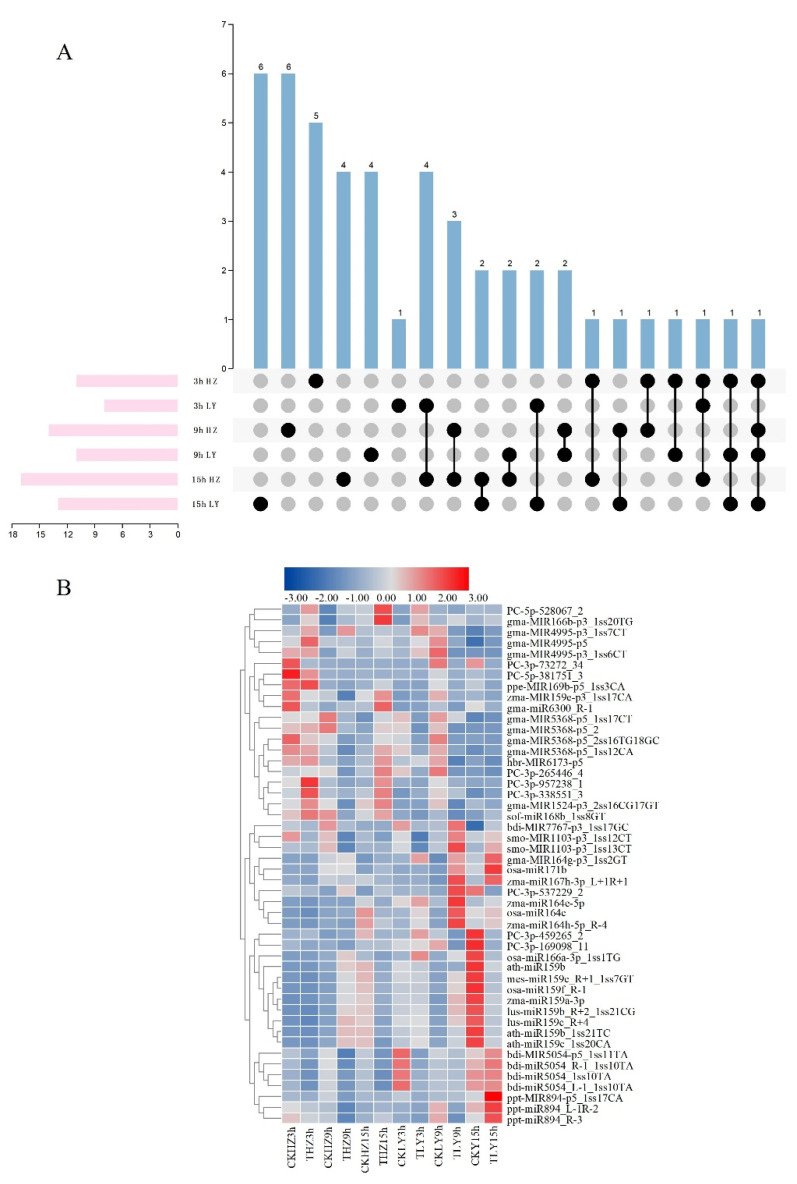
The characteristics of miRNAs identified to be related to disease resistant of maize to *C. lunata*. (**A**) Venn diagram of the number of miRNAs expressed differentially at different inoculation times in the susceptible variety HZ and the resistant variety LY. The “black dots” represent that the miRNAs could be expressed at the inoculation time point listed in the left and “grey dots” represent that the miRNAs could not be expressed at the inoculation time point listed in the left. (**B**) The expression dynamics of miRNAs involved in the disease resistant response of maize.

Through the combined analysis of high-throughput sequencing, microarray hybridization and degradome, 48 miRNAs were identified to be related to the resistance of maize to *C. lunata* (Table S13). To further investigate the association between *C. lunata*-responsive miRNAs and their target genes, interaction network analysis was performed using the Cytoscape platform (Figure 7). Among the 48 miRNAs, 14 were differentially expressed in both susceptible and resistant cultivars (Figure 8A and Table S13) and their expression patterns were different in HZ and LY. For example, at 3 hpi, the expression of PC-3p-957238_1 was up-regulated in HZ, but down-regulated in LY (Figure 8B). Furthermore, we found that some miRNAs could cleave different mRNA targets (Table S13), for example, PC-3p-265446_4 was found to be paired with transcripts “GRMZM2G056252” and “GRMZM2G011588”, which were annotated as “fatty acid desaturase 2” and “BEL1-like homeodomain 7”, respectively.

**Figure 9:**
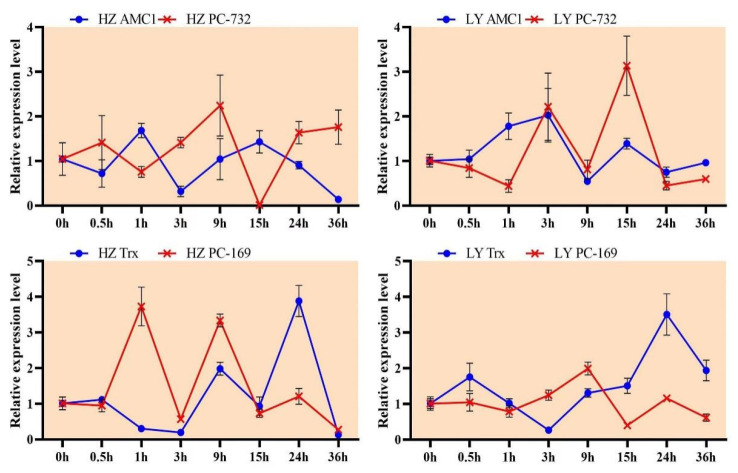
The expression profile of two novel miRNAs (PC-732 and PC-169) and their corresponding targets (*AMC*1 and *Trx*) in HZ (**left**) and LY (**right**).

The expression profiles of two novel miRNAs (PC-732 and PC-169) and their corresponding targets (*AMC*1 and *Trx*) were further investigated, and the HZ and LY samples were collected at 0, 0.5, 1, 3, 9, 15, 24 and 36 hpi. In the resistant variety LY, the expression of PC-732 and *AMC*1 showed negative correlation at the early inoculation stage (0–1 hpi): the expression of PC-732 was down-regulated, and *AMC*1 was up-regulated (Figure 9), indicating that *AMC*1 might take part in the regulation of early stage of disease resistance. However, in the susceptible variety HZ, the negative correlation mode between PC-732 and *AMC*1 was not obvious, speculating that PC-732 might not regulate the expression of *AMC*1 in HZ (Figure 9).

While the negative regulation between PC-169 and *Trx* were both obvious in LY and HZ. In the resistant variety LY, the expression of PC-169 was down-regulated and *Trx* was up-regulated from 15 hpi to 24 hpi (Figure 9). In other words, at the late stage of infection in LY, the expression of PC-169 was low, and the inhibition effect to *Trx* weakened, resulting in the high level of *Trx* expression and disease resistance. In susceptible variety HZ, PC-169 was highly expressed and strongly inhibited the expression of *Trx* at 1 hpi, and the inhibition effect of PC-169 to *Trx* had always occurred in HZ, leading to the disease occurrence (Figure 9). Following the results above, we concluded that PC-732 and PC-169 might take part in the disease resistant response of maize to *C. lunata*.

### 2.5. Knocking Down PC-732 Decreases Susceptibility of Maize to C. lunata

**Figure 10:**
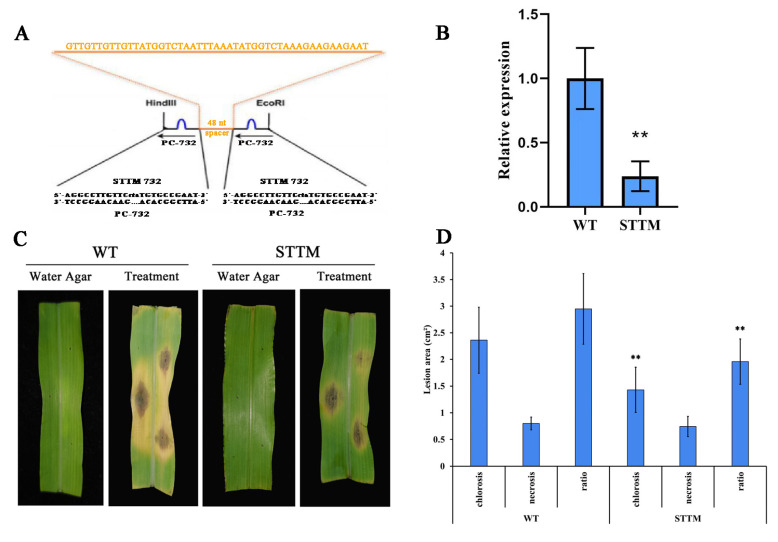
Knocking down PC-732 decreases susceptibility of maize to *C. lunata*. (**A**) The inhibitory expression vector STTM732 constructed through STTM (short tandem target mimic) technology. (**B**) The expression of PC-732 in transgenic plants were tested through stem-loop RT-PCR. “**” means significant difference (*p* < 0.01) between transgenic plants (STTM) and wildtype (WT). (**C**) The maize plants (WT and STTM) were inoculated by mycelium plugs of *C. lunata* and plugs of water agar were used as control. (**D**) Lesion area of chlorosis and necrosis on maize leaf (WT and STTM) inoculated by *C. lunata* were measured by Image J and ratio of chlorosis to necrosis was calculated. “**” means significant difference (*p* < 0.01) between STTM and WT.

To further confirm the role of PC-732 in the interaction of maize and *C. lunata*, transgenic plants in which the PC-732 was silenced by STTM were generated in maize B104, which is susceptible to *C. lunata* (Figure 10A) [29]. Stem-loop RT-PCR showed that the expression of PC-732 was suppressed in the transgenic plants (STTM) (Figure 10B). The wildtype (WT) and transgenic plants (STTM) were inoculated by *C. lunata*, and we found that the lesion area of necrosis showed no difference between STTM and WT, however, the lesion area of chlorosis for STTM was significantly smaller than WT and so was the ratio of chlorosis to necrosis (Figure 10C,D). The results indicated that knocking down PC-732 decreased susceptibility of maize to *C. lunata*, suggesting that PC-732 might inhibit the expression of disease resistance related genes during the interaction between maize and *C. lunata*.

## 3. Discussion

In this study, four sRNA libraries (ltR1, ltR2, ltR3 and ltR4) were generated from susceptible and the resistant varieties of maize inoculated by *C. lunata* and high-throughput sequenced. In the four libraries, the distribution of the length of sRNAs showed an uneven pattern. Among these, the 24 nt sRNAs were the most abundant, accounting for more than 1/4 of the total number of unique sequences in ltR1, ltR2 and ltR4. The finding was in accordance with the previous studies in maize [30] and other plants, such as *Medicago truncatula* [31] and potato [32]. Furthermore, the number of 24 nt sRNAs in ltR1 and ltR2 or ltR3 and ltR4 exhibited great differences, implying that the expression of sRNAs is responsive to *C. lunata* infection in maize.

The results also showed that the number of miRNAs in the four sRNA libraries were different. In ltR2, the number exhibited a downward trend after the susceptible variety HZ was inoculated with *C. lunata* compared with the control ltR1. However, when the resistance variety LY was infected by *C. lunata*, the number of miRNAs was much higher in ltR4 than in the control ltR3. Furthermore, based on the result of Venn diagram, ltR4 has the largest number of unique miRNAs. The results indicate that many miRNAs were induced in the resistant cultivar LY by *C. lunata* and might play an important role in resistant response to *C. lunata*.

In total, 72 novel miRNAs were identified in this study, and most of the novel miRNAs presented an induced and specific expression pattern, often with a low expression level. Similar results were published previously. Most of the new miRNAs were up- or down-regulated in response to the cadmium (Cd^2+^) exposure in rice [33]. And, the novel miRNAs identified through deep sequencing in *Brassica rapa* were all expressed in different tissues, but the expression level was low [34]. In addition, 3301 sRNAs that matched the maize genome but not found in miRBase were considered to be as potential novel miRNAs. These potential novel miRNAs increased the richness of miRNAs in maize and were good candidates for the study of disease resistance.

Microarray chip technology can be effectively used to find out differentially expressed miRNAs in plants. Dozens of soybean mosaic virus (SMV)-responsive miRNAs were identified in soybean by microarray analysis, and it was found that miR1507a, miR1507c and miR482a putatively regulated the expression of coding genes of NBS-LRR family proteins, which were related to the disease resistance of plants [35]. In this article, the expression levels of 1079 miRNAs at 3, 9 and 15 hpi in the susceptible variety HZ and the resistant variety LY were detected through microarray. The number of miRNAs responsive to *C. lunata* at the inoculation time points was different, being the highest at 9 hpi for HZ and at 15 hpi for LY. According to the tissue observation of maize infected by *C. lunata*, the pathogen germinated at 3 hpi, reached the infection point at 9 hpi and began to infect at 15 hpi, which was consistent with the conclusion published previously [36]. Therefore, the miRNAs that were expressed differentially at different inoculation time points might play a key role in the disease response of maize to *C. lunata*.

To figure out the functional importance of identified miRNAs, the degradome sequencing was performed to search their regulated targets. Previously, a total of 52 target mRNAs of 27 different miRNA families were identified in *P. patens* through degradome analysis, and many targets encoded putative regulatory proteins [21]. A total of 177 transcripts targeted by a total of 87 unique miRNAs were identified in *O. sativa* L. ssp. *Indica* using high-throughput degradome sequencing, and for the targets of conserved miRNAs between *Arabidopsis* and rice, 70% were transcription factors, indicating that these miRNAs act as masters of gene regulation in rice [37]. Using the same strategy, the targets of 112 conserved miRNAs and 44 novel miRNAs were identified in grapevine [24]. In this work, we generated two degradome libraries from susceptible variety (HZ) and resistant variety (LY), and totally identified 1584 targets cleaved by 665 miRNAs.

In addition, a total of 72 miRNA families were identified through miRNA family analysis, of which miR159, miR166 and miR156 were the most abundant. We found that members of the same miRNA family always showed similar expression trends through microarray analysis, which was consistent with reports published previously [38]. For example, the expression of some members of miR159, miR166 and miR6300 showed the trend of “first increase and then decrease” (ID), and some of miR5368 showed “first decrease and then increase” (DI) in HZ. In LY, the expression trend of some members of miR159 was “ID”. Meanwhile, some miRNA families, for example miR156, miR160 and miR166, shared conserved sequences and target genes, which was also discovered in other plants, from ferns to flowering plants [39,40]. The family miR-482/2118 showed special regulatory effects on NBS-LRR, defense genes during pathogen infection in plants [41,42]. The targets of miR164 were the transcript factor family NAC playing important roles in disease and stress resistance, growth and development [43,44,45].

Through the combined analysis of microarray and degradome sequencing, 48 miRNAs were screened out which might be related to disease resistance of maize to *C. lunata*. For example, the target of zma-MIR159e-p3_1ss17CA was VQ motif-containing protein, which involved in the regulation of a transcript factor WRKY [46]; the target of bdi-miR5054_1ss10TA was BAX inhibitor 1, which regulated death of cell [47]; zma-miR164h-5p_R-4 regulated several resistant-related genes, including cinnamate-4-hydroxylase, NAC domain containing protein 46 [48], Ankyrin repeat family protein [49] and myb domain protein 62 [50]. Furthermore, GRAS transcription factor, which was the target of osa-miR171b and is in the plant-specific transcription factor gene family, is involved in several developmental processes, phytohormone and phytochrome signaling, symbiosis, stress responses, etc. [51]. The UDP-glycosyltransferase (UGT) superfamily, which was regulated by the miRNA PC-5p-528067_2, catalyzed conjugation of small lipophilic compounds with sugars is an important detoxification and homeostatic function in all living organisms, including plants [52].

PC-169 and PC-732 were two novel miRNAs and were predicted to regulate the coding genes of thioredoxin (*Trx*) and apoptosis protein metacaspase 1 (*AMC*1), respectively. Depending upon the results of stem-loop RT-PCR, PC-732 and PC-169 negatively regulated the expression of their corresponding target genes. Thioredoxins (*Trx*) were closely related to the scavenging of reactive oxygen, therefore possibly participating in disease resistance of plants [53]. *AMC*1 was reported previously that could enhance the resistance of tobacco to *Colletotrichum destructivum* [54] and two types of metacaspase I (*AtMC*1 and *AtMC*2) were found in *A. thaliana*, both involved in disease resistance through positively regulating PCD [55]. In this article, we found that the lesion area of chlorosis on the transgenic plants was significant smaller than WT. It was reported that symptoms of the disease caused by *C. lunata* in maize included halo-surrounded lesions (chlorosis) partly due to toxin production [56]. Therefore, we speculated that silencing PC-732 might inhibit the synthesis of toxin of *C. lunata* or degrade the toxin synthesized by *C. lunata*, indicating that *AMC*1 possibly could improve the resistance of maize to *C. lunata*. The function of *AMC*1 in the interaction between maize and *C. lunata* need to be further explored.

## 4. Materials and Methods

### 4.1. Plant Materials

Two maize inbred lines were used in this experiment, Huangzao 4 (susceptible to *C. lunata*, hereinafter referred to as “HZ”) and Luyuan (highly resistant to *C. lunata*, hereinafter referred to as “LY”), which were kindly provided by Professor Chunsheng Xue (Shenyang Agricultural University, Shenyang, China). The seeds of HZ and LY were surface sterilized with 10% NaClO for 8 min and washed three times with sterile distilled water. Then the seeds were placed in an incubator at 28 °C (16 h L/8 h D) to germinate. Three seedlings per plastic pot, containing a mixture of sterile peat and sand, were planted, and thirty seedlings in total for each variety. The plants were irrigated with distilled water when needed.

### 4.2. Pathogen Inoculation

*C. lunata* strain CX-3, provided by Professor Jie Chen, Shanghai Jiaotong University, was cultured on potato dextrose agar (PDA) at 28 °C in darkness for 7 days. The conidia were collected using the solution containing 2% sucrose and 0.02% Tween 20 and the conidia suspension (10^6^ spore/mL) was sprayed onto the 7-leaf maize plants with an air sprayer. The plants sprayed with distilled water containing 2% sucrose and 0.02% Tween 20 were served as control. Fifteen plants were used for each treatment. All plants were divided into four groups. The first group (ltR1) was HZ control, the second group (ltR2) was HZ treated with *C. lunata* CX-3, the third group (ltR3) was LY control and the fourth group (ltR4) was LY treated with *C. lunata* CX-3. The 4th leaf of each group was harvested at 3, 9 and 15 h post inoculation (hpi), respectively, and five leaves for each inoculation time point. The samples collected from ltR1 at 3, 9 and 15 hpi were labeled as CKHZ3-A, CKHZ9-A and CKHZ15-A, respectively; the ones from ltR2 were labeled as THZ3-A, THZ9-A and THZ15-A, respectively; the ones from ltR3 were labeled as CKLY3-A, CKLY9-A and CKLY15-A, respectively; and the ones from ltR4 were labeled as TLY3-A, TLY9-A and TLY15-A, respectively. The samples were immediately frozen in liquid nitrogen and stored at −80 °C for total RNAs extraction, small RNAs sequencing, microarray hybridization, degradome sequencing and qRT-PCR.

### 4.3. Small RNA Libraries Construction, High-Throughput Sequencing and Data Analysis

The leaves collected at different inoculation time points of each group were sent to LC-Bio (Hangzhou, China) for sRNA libraries construction. The total RNAs were extracted using Trizol reagent (Invitrogen, Carlsbad, CA, USA) according to the manufacturer’s instructions, and the ones of samples collected at different time points (3, 9 and 15 hpi) from the same treatment were mixed in equal. Approximately 1 μg of total RNAs were prepared for the construction of four sRNA libraries (ltR1, ltR2, ltR3 and ltR4) following the guide of TruSeq Small RNA Sample Preparation kit (Illumina, San Diego, CA, USA). The purified cDNA libraries generated from RNA samples were used for cluster generation on Illumina’s Cluster Station and then sequenced on Illumina GAIIx according to vendor’s instructions. Raw reads sequenced were obtained using Illumina’s Sequencing Control Studio software, Version 2.8 (SCS v2.8) following real-time sequencing image analysis and base-calling by Illumina’s Real-Time Analysis Version 1.8.70 (RTA v1.8.70). The sequence data were processed according to the previously reported method with modifications [57]. Briefly, the raw reads were filtered using Illumina pipeline filter (Solexa 0.3), and the adapter dimers, junk, low complexity, other non-coding RNAs (rRNA, tRNA, snRNA, snoRNA) and repeats were removed with an in-house program, ACGT101-miR v4.2 (LC Sciences, Houston, TX, USA). Unique sequence families with same sequence were generated by sorting raw sequencing reads.

To identify known miRNAs and novel 3p- and 5p- derived miRNAs in the four libraries, the unique sequences were aligned against pre-miRNA (MIR) and mature miRNA (miR) sequences of maize listed in miRBase 21.0 according to the ACGT-101 user’s manual. In the BLAST search one mismatch inside of the sequence and length variation at both 3′ and 5′ ends were allowed. The unique sequences mapping to maize miRNAs in hairpin arms were identified as known miRNAs. The unique sequences mapping to the other arm of known MIR hairpin opposite to the annotated mature miRNA-containing arm were considered as novel 5p- or 3p derived miRNA candidates. For the remaining sequences, if they were mapped to the precursors of other selected species in miRBase 21.0, and the mapped MIRs were further aligned with the genome of maize to determine their genomic locations, they were defined as known miRNAs. The unmapped sequences were BLASTed against the maize genome, and the sequences containing RNA hairpin structures were predicated from the flank 120 nt sequences using RNAfold software (http://rna.tbi.univie.ac.at/cgi-bin/RNAWebSuite/RNAfold.cgi) (accessed on 12 October 2021). The criteria for secondary structure prediction were: (1) number of nucleotides in one bulge in stem (≤12); (2) number of base pairs in the stem region of the predicted hairpin (≥16); (3) cutoff of free energy (kCal/mol ≤ 15); (4) length of hairpin (up and down stems + terminal loop ≥ 50); (5) length of hairpin loop (≤200); (6) number of nucleotides in one bulge in mature region (≤4); (7) number of biased errors in one bulge in mature region (≤2); (8) number of biased bulges in mature region (≤2); (9) number of errors in mature region (≤4); (10) number of base pairs in the mature region of the predicted hairpin (≥12); (11) percent of mature in stem (≥80).

### 4.4. MiRNA Microarray Assay

Microarray assay was performed by a service provider, LC Sciences (Houston, TX, USA) according to the company’s protocols. 4–8 μg total RNAs of different samples (CKHZ3-A, CKHZ9-A, CKHZ15-A, THZ3-A, THZ9-A, THZ15-A, CKLY3-A, CKLY9-A, CKLY15-A, TLY3-A, TLY9-A and TLY15-A) were used. The microfluidic chip probes contained 1079 miRNAs from 43 species. The probes designed according to maize 5SrRNAs were used as internal positive controls, synthetic probes were external positive controls, and blank and non-homologous nucleic acids were negative controls (Table S1). There were three technical replicates for each treatment.

The detection probes were made by in situ synthesis using photogenerated reagent (PGR) chemistry. The hybridization melting temperatures were balanced by chemical modifications of the detection probes. Hybridization was performed at 34 °C in 100 μL 6 × SSPE buffer [0.90 M NaCl, 60 mM Na_2_HPO_4_, 6 mM ethylenediaminetetraacetic acid (EDTA), pH6.8] plus 25% formamide. The complex of Cy3-labeled RNA and probe was dyed through circulation in the microfluidic chip. Fluorescence images were collected using a laser scanner (GenePix 4000B, Molecular Device, Sunnyvale, CA, USA) and digitized using image analysis software Array-Pro (Media Cybernetics, Rockville, MD, USA). After the background was subtracted, data were normalized using LOWESS filter (locally-weighted regression) [58] and then cluster analyzed using Cluster 3.0 (Stanford University) to get the ratio of detection signal of treatment to control (or resistant variety LY to susceptible variety LY) and *p*-value in *t*-test. *p*-value < 0.01 and ∣log_2_ fold change∣> 1.5 were defined as the threshold of the differentially expressed miRNAs [59,60,61]. The miRNAs differentially expressed were chosen to draw a heatmap with MultiExperiment Viewer Version 4.0, and a clustering analysis using the hierarchical clustering method was performed [62].

### 4.5. Degradome Library Construction, Sequencing and Analysis

The degradome libraries of HZ and LY were constructed by a service provider (LC Sciences, Houston, TX, USA). The total RNAs were extracted using Trizol reagent (Invitrogen, CA, USA) according to the manufacturer’s instructions. The quantity and purity of total RNAs were analyzed using Bioanalyzer 2100 and RNA 6000 Nano LabChip Kit (Agilent, Palo Alto, CA, USA) with RIN number >7.0. Approximately 20 μg of total RNAs were prepared for the construction of the Degradome library [63]. The purified cDNA libraries were used for cluster generation on Illumina’s Cluster Station and then the single-end sequencing (36 bp) was performed on an Illumina Hiseq2500 following the vendor’s recommended protocols. Raw sequencing reads were obtained using Illumina’s Pipeline v1.5 software following sequencing image analysis by the Pipeline Firecrest Module and base-calling by the Pipeline Bustard Module. The mappable sequences were analyzed with the software package CleaveLand 3.0 [64] and blasted with maize cDNA database to generate degradome density file. The target mRNA sequences paired with miRNAs were predicted by the software TargetFinder. The degradome density file was compared to the target predictions to find out the common mRNAs, which were the targets of miRNAs [65]. The annotations of candidate target genes were performed using the Blast2 GO Gene Ontology Functional Annotation Suite (GO, http://www.geneontology.org/) (accessed on 25 October 2021) and the Kyoto Encyclopedia of Genes and Genomes (KEGG). There are three biological replicates for each library.

### 4.6. Expression Pattern of MiRNAs and Their Target mRNAs Using Stem-Loop RT-PCR

To test if the expression pattern of miRNA and its counterpart target gene were in negative correlation, stem-loop real-time PCR (stem-loop RT-PCR) was performed. Samples of HZ and LY inoculated by *C. lunata* were collected at different inoculation time points and their total RNAs were extracted as described earlier. Reverse transcription PCR (RT-PCR) was performed using PrimeScript^TM^ RT Kit (Takara, Dalian, China), and the specific stem-loop RT primer for miRNAs (Table S2) and the oligo dT primer for target mRNAs were used. cDNAs were diluted 20-fold with sterile water before being used as a template in qRT-PCR which was performed on ABI 7500 (Applied Biosystems, Carlsbad, CA, USA) with SYBR Premix Ex Taq™ II (Takara, Japan) according to the standard protocol. The reverse and forward primers for all selected miRNAs and targets are available in Table S2. The miR172 and GADPH gene of maize were used as internal reference of miRNA and target gene, respectively. Three replicates were performed for each treatment.

### 4.7. The Combined Analysis

To find the miRNAs that were related to the disease resistance of maize to *C. lunata*, the combined analysis of high-throughput sequencing, microarray hybridization and degradome was performed. First, four sRNA libraries were constructed, and miRNAs were obtained through high-throughput sequencing. Second, through microarray analysis, the miRNAs that were differentially expressed were selected at each inoculation time in the susceptible variety HZ and the resistant variety LY (*p*-value of *t*-test < 0.01 and log_2_ fold change∣ > 1.5). Third, the target genes of miRNAs that were differentially expressed were confirmed through degradome sequencing or were predicted through TargetFinder. Lastly, according to the function annotation and referring to the articles published previously, the target genes relative to disease resistance were identified, and the miRNAs paired to these target genes were our candidates.

### 4.8. The Function Analysis of PC-732

To test if PC-732 was related to the disease resistance of maize, transgenic plants were generated where PC-732 expression was inhibited. To fulfill the goal, the inhibitory expression vector STTM732 was constructed through STTM (short tandem target mimic) technology [29], integrated into pCAMBIA3301 and transformed into maize B104 [66]. Transgenic plants were selected by resistance to the herbicide BASTA and tested through stem-loop RT-PCR (primers used referred to Table S2).

To check the disease resistance of transgenic plants, the leaves were surface-sterilized with cotton balls soaked by 75% alcohol, wounded with a sterilized needle, then inoculated with 5 mm mycelia plugs of *C. lunata* cultured on PDA at 28 °C for 7 days. The petioles of the inoculated leaves were wrapped with wet cotton balls to keep the leaves moisturized and placed in plastic boxes covered with wet gauze. The leaves inoculated with water agar were used as control. The boxes were incubated at 28 °C for 4 days and the disease incidence was observed. The software Image J (Wayne Rasband, National Institute of Health, Bethesda, MD, USA) was used to measure the lesion area.

## 5. Conclusions

A total of 2286, 2145, 1556 and 2504 miRNAs were identified in the four sRNA libraries, ltR1, ltR2, ltR3 and ltR4, respectively, which were generated from the susceptible variety Huangzao 4 (HZ) and the resistant variety Luyuan (LY) of maize inoculated by *C. lunata*. Through the combined analysis of high-throughput sequencing, microarray hybridization and degradome, 48 miRNAs were identified as being related to maize resistance to *C. lunata*. Among these, PC-732 and PC-169, two new maize miRNAs discovered, were predicted to cleave mRNAs of metacaspase 1 (*AMC*1) and thioredoxin family protein (*Trx*), respectively, possibly playing crucial roles in the resistance of maize to *C. lunata*. Furthermore, knocking down PC-732 decreased the susceptibility of maize to *C. lunata*, and the target gene of PC-732 might inhibit the expression of disease-resistance related genes during the interaction between maize and *C. lunata*.

## Figures and Tables

**Table 1:** Statistical data of sRNAs sequences in resistant, susceptible and control libraries.

Category	ltR1		ltR2		ltR3		ltR4	
	Unique sRNAs	Total sRNAs	Unique sRNAs	Total sRNAs	Unique sRNAs	Total sRNAs	Unique sRNAs	Total sRNAs
Raw reads	1,050,117 (100%)	7,899,745 (100%)	910,208 (100%)	6,443,535 (100%)	848,756 (100%)	7,508,744 (100%)	1,009,022 (100%)	7,250,729 (100%)
3ADT&length filter	454,446 (43.28%)	2,365,487 (29.94%)	429,846 (13.06%)	2,479,058 (38.47%)	451,091 (53.15%)	2,916,553 (38.84%)	459,048 (45.49%)	2,685,086 (37.03%)
Junk reads	3778 (0.36%)	6352 (0.08%)	2916 (0.32%)	4807 (0.07%)	2655 (0.31%)	6630 (0.09%)	3535 (0.35%)	5913 (0.08%)
Rfam	64,914 (6.18%)	886,230 (11.22%)	55,806 (6.13%)	719,210 (11.16%)	48,798 (5.75%)	805,644 (10.73%)	55,694 (5.52%)	919,838 (12.69%)
mRNA	76,579 (7.29%)	324,059 (4.10%)	70,179 (7.71%)	327,289 (5.08%)	67,498 (7.95%)	325,434 (4.33%)	84,851 (8.41%)	323,200 (4.46%)
Repeats	2855 (0.27%)	21,889 (0.28%)	2850 (0.31%)	19,085 (0.3%)	2923 (0.34%)	29,134 (0.39%)	2452 (0.24%)	25,263 (0.35%)
miRNA	2286 (0.22%)	71,161 (0.9%)	2145 (0.24%)	76,375 (1.19%)	1556 (0.18%)	47,352 (0.63%)	2504 (0.25%)	79,071 (1.09%)
Clean reads	453,404 (43.18%)	4,294,732 (54.37%)	354,557 (38.95%)	2,888,230 (44.82%)	282,051 (33.23%)	3,444,122 (45.87%)	408,151 (40.45%)	3,351,119 (46.22%)
other Rfam RNA	6034 (0.08%)	85,212 (1.08%)	5018 (0.08%)	78,366 (1.22%)	5081 (0.07%)	127,599 (1.70%)	4684 (0.06%)	90,218 (1.24%)

**Table 2:** Overview of reads from degradome sequencing.

Sample	HZ (Number)	HZ (Ratio)	LY (Number)	LY (Ratio)	Sum (Number)	Sum (Ratio)
Raw reads	11,519,270	/	14,012,080	/	25,531,350	/
Reads < 15 nt after removing 3′ adaptor	45,342	0.39%	50,584	0.36%	95,926	0.38%
Mappable reads	11,473,928	99.61%	13,961,496	99.64%	25,435,424	99.62%
Unique raw reads	3,831,009	/	3,571,729	/	6,472,505	/
Unique reads < 15 nt after removing 3′ adaptor	21,354	0.56%	19,150	0.54%	35,779	0.55%
Unique mappable reads	3,809,655	99.44%	3,552,579	99.46%	6,436,726	99.45%
Transcript mapped reads	9,517,279	82.62%	11,676,151	83.33%	21,193,430	83.01%
Unique transcript mapped reads	2,996,086	78.21%	2,864,461	80.20%	5,017,136	77.51%
Number of input transcript	88,760	/	88,760	/	88,760	/
Number of covered transcript	64,926	73.15%	65,140	73.39%	69,774	78.61%

## Data Availability

Raw sequencing data generated in this study have been submitted to the NCBI BioProject database (http://www.ncbi.nlm.nih.gov/bioproject/) (accessed on 9 October 2021) under accession number: PRJNA769949.

## Supplementary Materials

The following supporting information can be downloaded at: https://www.mdpi.com/article/10.3390/ijms232214038/s1.

## Abbreviations

miRNAs	microRNAs
sRNA	small RNA
RNAi	RNA interference
stem-loop RT-PCR	stem-loop real-time PCR
QTL	quantitative trait locus
nt	nucleotide
hpi	hours post inoculation
GO	Gene Ontology
ATP	adenosine triphosphate
DNA	deoxyribonucleic acid
mRNA	messenger RNA
SMV	Soybean Mosaic Virus
UDP	Uridine diphosphate
UGT	glycosyltransferase
PGR	photogenerated reagent
RT-PCR	Reverse transcription PCR
qRT-PCR	quantificational real-time PCR
STTM	Short Tandem Target Mimic
