# Supplementary material for: Integrated High-Throughput Sequencing, Microarray Hybridization and Degradome Analysis Uncovers MicroRNA-Mediated Resistance Responses of Maize to Pathogen Curvularia lunata
Source: Int J Mol Sci. 2022 Nov 14;23(22):14038. doi: 10.3390/ijms232214038 (PMC9697682; doi:10.3390/ijms232214038)
Supplement: Supplementary file 1 [file ijms-23-14038-s001.zip › supplementary files/Supplementary Figures 1¿C3.pdf]

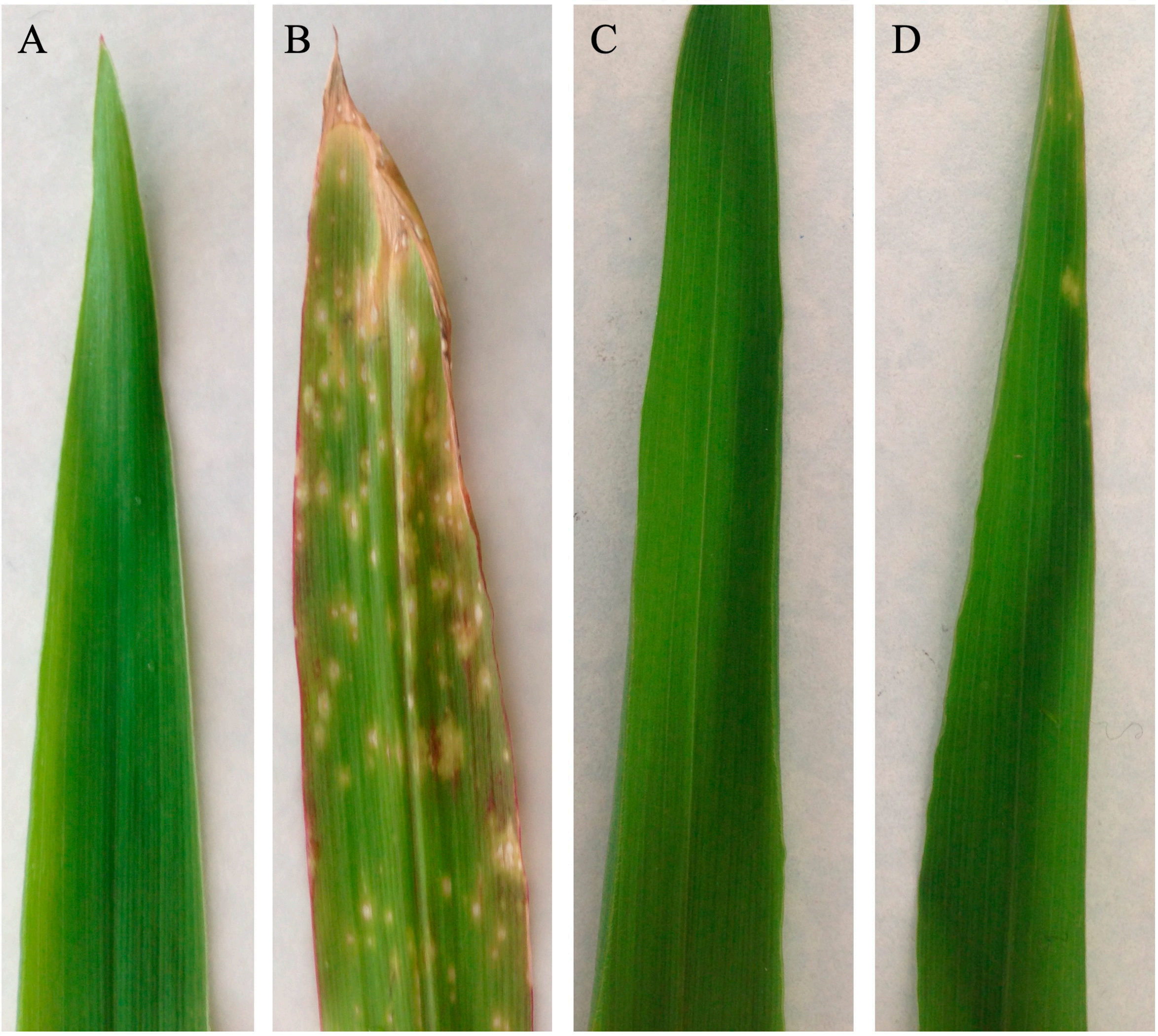

Figure S1. Symptom of *C. lunata* on the susceptible variety HZ and variety cultivar LY. (A) HZ not inoculated by *C. lunata*. (B) HZ inoculated by *C. lunata*. (C) LY not inoculated by *C. lunata*. (D) LY inoculated by *C. lunata*.

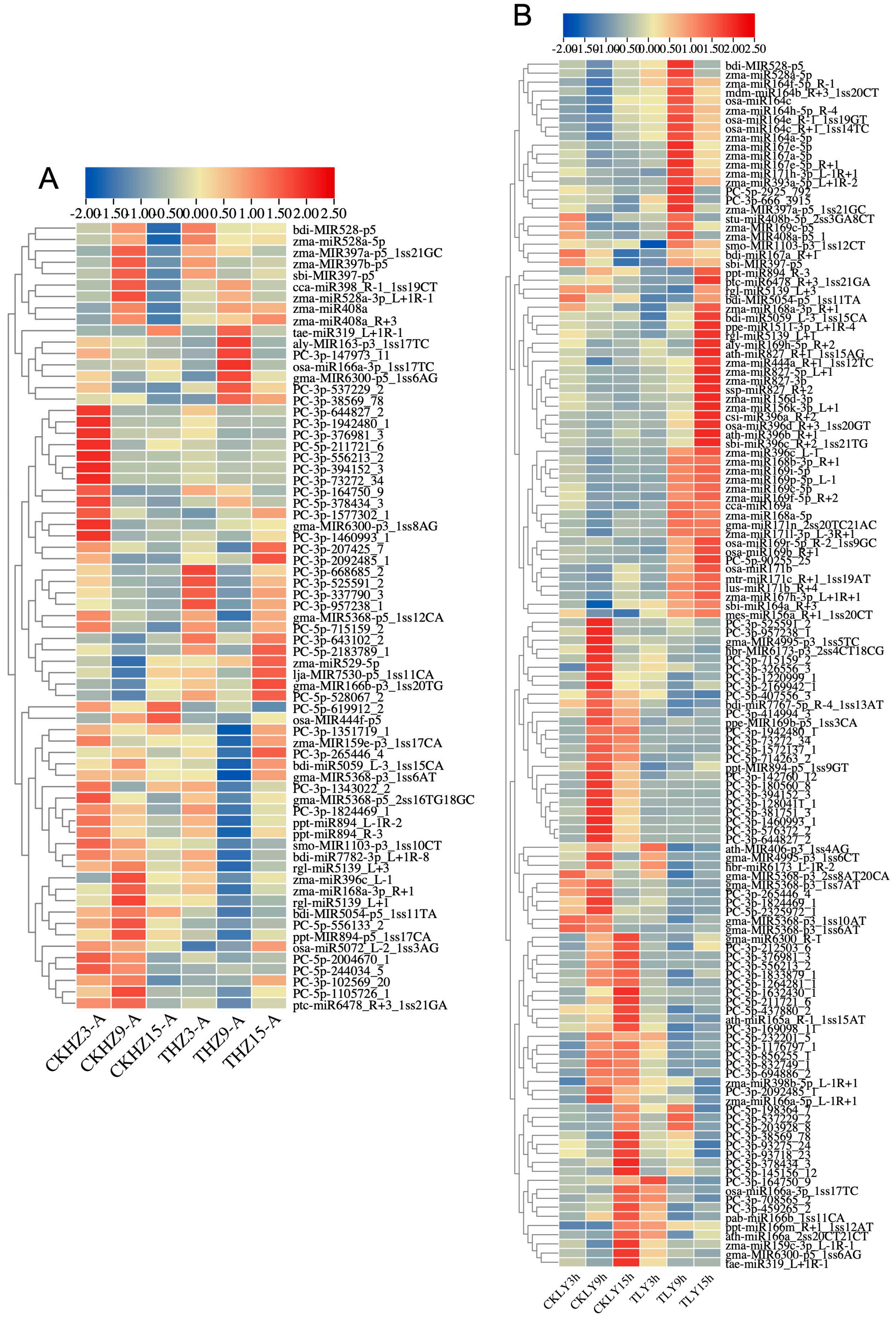

Figure S2. The miRNAs whose expression levels changed significantly in the susceptible variety HZ (A) and the resistant variety LY (B).

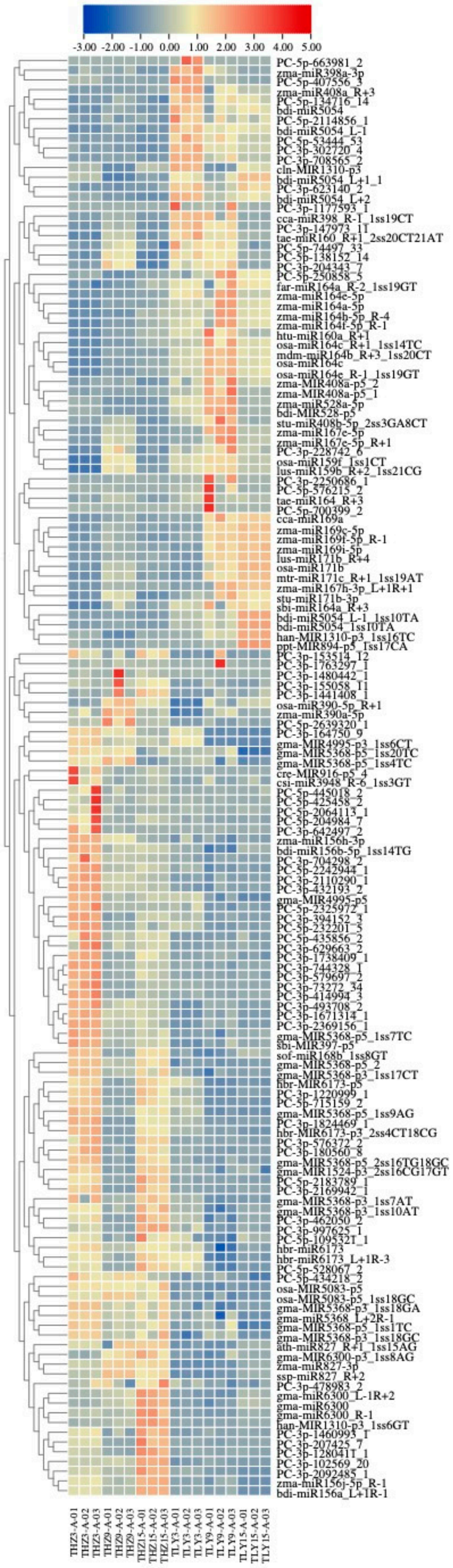

Figure S3. Expression dynamics of miRNAs that expressed differentially in the resistant variety LY compared with the susceptible variety HZ.
